# Pilot Study of Physical and Psychosocial Health Outcomes and Caregiver Burden in Mothers of Children with Physical Disabilities in Türkiye: A Cross-Sectional Analysis

**DOI:** 10.3390/healthcare14050632

**Published:** 2026-03-02

**Authors:** Ebrar Atak, Erdi Kayabınar, Büşra Kayabınar, Fatma Mutluay

**Affiliations:** Department of Physiotherapy and Rehabilitation, Faculty of Health Sciences, Yalova University, Yalova 77200, Türkiye; erdi.kayabinar@yalova.edu.tr (E.K.); busra.kayabinar@yalova.edu.tr (B.K.); fatma.mutluay@yalova.edu.tr (F.M.)

**Keywords:** caregiver burden, mothers, physical disability, depression, psychosocial health

## Abstract

**Highlights:**

**What are the main findings?**

- Depressive symptoms and functional limitations were consistently associated with lower quality-of-life scores in caregiving mothers.
- Higher physical activity was associated with better vitality, social functioning, and mental health.

**What are the implication of the main findings?**

- Physiotherapy and psychosocial programs may be relevant for supporting mental and physical health in mothers who have children with physical disabilities.
- Encouraging active lifestyles in caregivers may be associated with lower long-term physical and psychological burden.

**Abstract:**

**Background:** Mothers caring for children with physical disabilities experience sustained physical and psychological demands; however, evidence simultaneously examining physical activity, functional limitation, pain, caregiver burden, and mental health within local caregiving contexts remains limited. **Objective:** This pilot study aimed to explore the multidimensional associations between physical and psychological health outcomes and health-related quality of life in mothers caring for children with physical disabilities in Türkiye. **Methods:** Forty volunteer mothers residing in Yalova, Türkiye, were assessed using the IPAQ–SF, BDI, SF-36, ZBI, ODI, DASH, and SF-MPQ. Data were analyzed in SPSS 26.0 using correlation analyses and exploratory multiple linear regression models, with *p* < 0.05 considered statistically significant. Descriptive statistics, Pearson or Spearman correlation analyses, and exploratory multiple linear regression models were applied. **Results:** The mean age was 38.52 ± 9.10 years. Depression (BDI) and functional limitation showed pronounced negative associations with quality-of-life domains (e.g., General Health: r = −0.749, *p* < 0.001). Moderate physical activity was associated with higher vitality, social functioning, and mental health (*p* < 0.05). **Conclusions:** The findings suggest co-occurring links between psychological distress and physical functioning in caregiving mothers. Within the exploratory scope of this pilot study, multidisciplinary approaches that integrate physiotherapy and psychosocial support may be relevant for supporting caregiver health needs.

## 1. Introduction

Raising a child with physical disabilities entails sustained emotional and physical demands, particularly for mothers. Caregiving mothers carry continuous responsibilities that translate into multidimensional burdens across physical, psychological, social, and economic domains [1,2]. Studies have demonstrated that mothers of children with physical disabilities often experience elevated levels of depression, anxiety, stress and burnout, leading to a marked decline in their overall quality of life [2,3,4,5,6,7]. In Türkiye, caregiving responsibilities predominantly fall on mothers in socioeconomically constrained households, where limited formal and informal support further intensifies psychological distress and physical strain [8,9]. In many families in Türkiye, mothers shoulder the majority of daily care responsibilities for children with disabilities, including physical assistance, medical follow-up, and household management. Therefore, focusing solely on mothers reflects both the sociocultural structure of caregiving in Türkiye and the clinical significance of examining the subgroup most exposed to cumulative physical and psychological burdens.

From a physical health perspective, mothers of children with physical disabilities commonly report chronic musculoskeletal disorders, back and shoulder pain, sleep disturbances, and persistent fatigue, reflecting cumulative biomechanical strain and irregular caregiving schedules that promote physiological stress accumulation [5,6]. Repetitive childcare movements, suboptimal postural strategies, insufficient rest, and disrupted sleep patterns constitute the primary contributing factors [7]. Over time, sustained physical loading may contribute to chronic pain syndromes, osteoarticular degeneration, and progressive declines in functional capacity [8].

Psychologically, mothers of children with physical disabilities exhibit higher levels of stress and anxiety than the general population [2]. Continuous caregiving demands, limited personal time, social isolation, and financial strain collectively exacerbate depressive symptoms and burnout [4]. In particular, among mothers of physically disabled children with low socioeconomic status, restricted social support and financial hardship further weaken psychological well-being [1].

Restricted workforce participation may undermine economic independence; consequently, flexible employment arrangements and supportive welfare policies may partially mitigate the social and health-related consequences of long-term caregiving [7].

Caregiver health is multidimensional, spanning physical, psychological, and social dimensions. However, many studies still address these domains in isolation, leaving the joint patterning of physical activity, functional limitations, caregiver burden, depressive symptoms, pain, and health-related quality of life insufficiently characterized—particularly in local caregiving contexts where formal support may be limited. In this regard, strengthening access to psychosocial support and feasible activity-oriented strategies may be relevant for addressing co-occurring caregiver needs [2,4,9]. Empirical evidence remains limited regarding the integrated evaluation of physical activity, functional limitation, caregiver burden, depressive symptoms, and health-related quality of life within a single analytical framework, leaving the combined influence of these interrelated factors insufficiently characterized in local caregiving populations. Accordingly, this pilot study examines the multidimensional associations between physical activity level, depressive symptoms, caregiver burden, pain intensity, functional limitations, and health-related quality of life among mothers of children with physical disabilities in Türkiye. Unlike many caregiver quality-of-life studies that focus primarily on psychological distress or burden in isolation, the present study simultaneously integrates physical activity level, region-specific functional limitations (upper and lower extremity), pain intensity, depressive symptoms, and caregiver burden within a single analytical framework. This multidimensional integration enables a more clinically actionable understanding of how physical and psychological domains co-occur in caregiving mothers within a socioculturally specific Turkish perspective.

Accordingly, the study addresses the following research questions:

(1) What are the relationships between physical activity, depressive symptoms, caregiver burden, pain intensity, functional limitations, and SF-36 quality-of-life domains in caregiving mothers?
(2) Which clinical variables show the most consistent associations with health-related quality of life in this population?
(3) Do psychological and functional variables show differential patterns of association across specific quality-of-life subdomains?

As a pilot investigation, this study aims to map hypothesis-relevant co-occurring patterns and to inform the design and measurement strategy of subsequent adequately powered studies in similar caregiving contexts. Importantly, this study was designed as hypothesis-generating rather than hypothesis-testing, with primary emphasis on feasibility, pattern identification, and multidimensional measurement integration rather than on confirmatory statistical inference. We hypothesized that higher levels of depression and functional limitation would be associated with lower SF-36 quality-of-life scores, whereas higher physical activity levels would be associated with better vitality and mental health.

## 2. Materials and Methods

### 2.1. Study Design

This study was designed as a cross-sectional, correlational pilot study to examine multidimensional associations between caregiver mothers’ physical activity level, depressive symptoms, caregiver burden, pain intensity, and functional limitations, and their health-related quality of life. The primary outcomes were the SF-36 subscale scores, and the main explanatory variables were IPAQ-SF, BDI, ZBI, SF-MPQ, DASH/DASH-W, ODI, and age. A quantitative approach was selected to describe co-occurring patterns across biopsychosocial domains in a real-world caregiving situations, without implying directionality or causality [4,7]. Consistent with its pilot character, the analytical strategy prioritized descriptive feasibility assessment and exploratory pattern mapping, rather than formal hypothesis testing or model confirmation.

### 2.2. Participants

‘Sex’ refers to biological sex; gender-related roles were not assessed. Physical disability was defined as neuromuscular or musculoskeletal impairments that affect movement, posture, and functional capacity. Common examples include conditions such as cerebral palsy, spina bifida, and muscular dystrophy. Eligibility for ‘physical disability’ status was based on the child’s official Health Board or Guidance and Research Center report and confirmation of ongoing rehabilitation follow-up (as reported by the mother). The research was conducted with 40 volunteer mothers residing in Yalova, Türkiye, each of whom had at least one child with a physical disability. Participants were recruited through convenience sampling among volunteer mothers who met the inclusion criteria. No probabilistic sampling frame was used, and sampling error was not calculated due to the exploratory nature of the study.

Child-related variables such as age, sex, diagnosis, and severity of disability were not systematically recorded in this pilot phase. This was a deliberate design decision to maintain analytical focus on maternal physical and psychological burden and to reduce additional reporting complexity during feasibility testing. However, these variables may influence caregiving strain and will require structured documentation in future, larger-scale studies.

*Inclusion criteria:*

- Female participants aged 25 years or older (the lower age limit of 25 years was selected because this period represents adulthood in which the maternal role is fulfilled in a more physiologically and socially stable manner, and it coincides with the age range in which caregiving responsibilities are most intense) [10];
- Having at least one child with a physical disability;
- The child’s physical disability being documented by an official Health Board or Guidance and Research Center report. In Türkiye, these reports are official documents in which the medical and functional dimensions of disability are evaluated, and they are mandatory for accessing government-supported services;
- Voluntary participation through the signing of an informed consent form.

Participants with missing data were excluded from the final analysis; no data imputation or estimation methods were used.

### 2.3. Data Collection Instruments

To enhance readability and reduce redundancy, core measurement characteristics are summarized in Table 1, while detailed scoring procedures are reported in the original validation sources. The following standardized assessment tools were used to evaluate participants’ physical, psychological, and functional status:

*International Physical Activity Questionnaire—Short Form (IPAQ–SF)*

The IPAQ–SF assesses self-reported physical activity levels over the previous seven days [11]. The Turkish validity and reliability study was conducted by Saglam (2010) [12]. Scoring procedures and activity level classifications are summarized in Table 1.

*Short Form-36 Health Survey (SF-36)*

Developed by Ware et al. (1993), the SF-36 assesses health-related quality of life across eight subscales: physical functioning (PF), role limitations due to physical health (RP), role limitations due to emotional problems (RE), bodily pain (BP), general health (GH), vitality (VT), social functioning (SF), and mental health (MH) [13]. The Turkish validity and reliability of the SF-36 has been reported previously [14]. Scoring characteristics and subscale domains are summarized in Table 1.

*Beck Depression Inventory (BDI)*

Developed by Beck et al. (1961), this inventory measures the severity of depressive symptoms over the past two weeks [15]. The Turkish adaptation was validated by Durak & Palabıyıkoğlu (1994) [16]. Item structure and severity classification thresholds are summarized in Table 1.

*Zarit Caregiver Burden Interview (ZBI)*

Developed by Zarit, Reever, and Bach-Peterson (1980), the ZBI evaluates emotional, physical, social, and economic burden among caregivers [17]. The Turkish version was validated by İnci and Erdem (2010) [18]. Instrument structure and scoring range are summarized in Table 1.

*Oswestry Disability Index (ODI)*

Developed by Fairbank and Pynsent (2000), the ODI assesses the impact of low back pain on daily living activities [19]. The Turkish adaptation was conducted by Yakut et al. (2004) [20]. Disability scoring and severity categorization are summarized in Table 1.

*Disabilities of the Arm, Shoulder and Hand (DASH) Questionnaire*

Developed by Hudak et al. (1996), the DASH is a 30-item self-report tool used to evaluate upper extremity functional limitations and symptoms [21]. The Turkish adaptation was validated by Düger et al. (2006) [22]. Scoring procedures and interpretation ranges are summarized in Table 1.

*DASH–Work Module (DASH-W)*

The DASH–Work Module (DASH-W) is an optional 4-item module developed alongside the DASH to quantify work-related upper-limb limitations beyond general daily activities. In this study, it was used to capture functional restrictions relevant to caregiving mothers who may also perform household or occupational tasks requiring repetitive upper-extremity use [23]. Work-related scoring characteristics are summarized in Table 1.

*Short-Form McGill Pain Questionnaire (SF-MPQ)*

Originally developed by Melzack (1975), the McGill Pain Questionnaire evaluates sensory, affective, and evaluative dimensions of pain [24]. The Turkish adaptation was validated by Yakut et al. (2007) [25]. Pain scoring characteristics are summarized in Table 1.

**Table 1:** Summary of Measurement Instruments Used in the Study.

Instrument	Abbreviation	No. of Items	Measured Domain	Turkish Validation Study
International Physical Activity Questionnaire—Short Form	IPAQ-SF	7	Physical activity level	Saglam, 2010 [12]
Beck Depression Inventory	BDI	21	Depression severity	Durak & Palabıyıkoğlu, 1994 [16]
Short Form-36 Health Survey	SF-36	36	Quality of life (8 subscales)	Koçyigit et al., 1999 [14]
Zarit Caregiver Burden Interview	ZBI	22	Caregiver burden	İnci & Erdem, 2010 [18]
Oswestry Disability Index	ODI	10	Low back functional limitation	Yakut et al., 2004 [20]
Disabilities of the Arm, Shoulder and Hand	DASH	30	Upper extremity function	Düger et al., 2006 [22]
DASH–Work Module	DASH-W	4	Work-related upper limb function	House et al., 2012 [23]
Short-Form McGill Pain Questionnaire	SF-MPQ	15	Pain intensity and quality	Yakut et al., 2007 [25]

The combined assessment battery comprised 145 items across multiple validated instruments. While this comprehensive approach strengthened multidimensional construct coverage, it also introduced potential respondent burden. Administering a lengthy questionnaire set to caregiving mothers—who often experience time constraints, cumulative fatigue, and psychological strain—may influence concentration, attentional stability, and response consistency. During pilot feasibility testing, particular attention was given to administration setting, pacing, and the allowance of short breaks to mitigate fatigue-related effects. The estimated completion time for the full assessment was approximately 60–75 min, depending on literacy level and interruptions.

### 2.4. Statistical Analysis

The primary dependent variables were the SF-36 subscale scores. Normality was assessed using the Shapiro–Wilk test. Descriptive statistics were reported as mean ± standard deviation or median (minimum–maximum), as appropriate. Group comparisons by physical activity category (low vs. moderate) were performed using the Independent Samples *t*-test or Mann–Whitney U test according to distributional assumptions. Bivariate associations among clinical variables and SF-36 subscales were examined using Pearson or Spearman correlation analyses. In an exploratory and descriptive manner, multiple linear regression models were fitted with each SF-36 subscale as an outcome and age, BDI, ZBI, SF-MPQ, DASH, and ODI as covariates to map multivariable co-occurrence patterns. These models were not constructed for prediction, hypothesis confirmation, or generalizable inference, but solely to provide descriptive pattern exploration within a pilot framework. Given the limited sample size relative to the number of predictors, coefficient stability and model precision are inherently constrained. No formal correction for multiple comparisons was applied, confidence intervals were not systematically reported, and findings were therefore interpreted strictly as exploratory signals rather than statistically robust estimates. Statistical analyses were conducted in SPSS version 26.0 (IBM Corp., Chicago, IL, USA), with *p* < 0.05 considered statistically significant. Reporting was guided by the STROBE statement [26].

### 2.5. Ethical Approval and Participant Rights

This study was approved by the Ethics Committee for Non-Interventional Clinical Research of Yalova University (Approval No: 2025/87, dated 5 March 2025). Participants were informed about the study’s objectives, confidentiality principles, and voluntary participation rights. Written informed consent was obtained from all participants. All data were anonymized and used exclusively for scientific purposes. Participants retained the right to withdraw from the study at any time without any consequences.

## 3. Results

### 3.1. Demographic Characteristics and Clinical Assessment Findings

The study included 40 mothers of children with physical disabilities, with a mean age of 38.52 ± 9.10 years (range: 25–68 years). Regarding educational attainment, 40% of the participants were high school (HS) graduates, 30% had completed middle school (MS), and 30% had completed primary school (PS); no participants were illiterate or held a university degree, suggesting that the sample represented a low-to-moderate educational background. According to the results of the International Physical Activity Questionnaire–Short Form (IPAQ–SF), 62.5% of participants exhibited low physical activity levels, while 37.5% demonstrated moderate levels; no participants reported high physical activity. These findings indicate a generally sedentary lifestyle and low physical fitness among the participants. Regarding quality of life, the SF-36 subscale scores revealed the highest means in Social Functioning (75.10 ± 22.52) and Physical Functioning (69.62 ± 22.76), while the lowest were observed in Role-Emotional (32.45 ± 31.55) and Vitality (39.00 ± 17.10). This pattern suggests that although participants maintained a degree of social engagement, they experienced a marked decline in emotional resilience and energy levels.

The mean scores of psychological and functional assessment tools were as follows:

Beck Depression Inventory (BDI): 14.35 ± 9.70, Zarit Caregiver Burden Interview (ZBI): 48.4 ± 15.8, Short Form-McGill Pain Score (SF-MPQ): 29.3 ± 9.7, DASH: 21.91 ± 19.19, DASH–Work Module: 27.26 ± 26.73, and Oswestry Disability Index: 14.55 ± 15.13. These values indicate mild to moderate depressive symptoms, substantial caregiver burden, and generally moderate levels of pain and functional limitation among participants (Table 2).

### 3.2. Bivariate Associations Between Scales

Correlation analyses revealed statistically significant negative relationships between depression level (BDI) and several SF-36 quality of life subscales (*p* < 0.05). The strongest negative correlations were observed with General Health (r = −0.749, *p* < 0.001), Vitality (r = −0.685, *p* < 0.001), Social Functioning (r = −0.594, *p* < 0.001), and Mental Health (r = −0.635, *p* < 0.001). Overall, higher depressive symptom levels tended to coincide with higher depressive symptom levels tend to coincide with lower perceived general health, energy levels, and social functioning. Similarly, Zarit Caregiver Burden Interview (ZBI) was found to be negatively correlated with all SF-36 subscales, with the strongest correlations noted in Social Functioning (r = −0.604, *p* < 0.001) and Mental Health (r = −0.563, *p* < 0.001). This pattern suggests that higher caregiving burden is associated with lower quality of life and reduced social participation. Furthermore, McGill Pain, DASH, and ODI scores demonstrated significant negative correlations with selected SF-36 subscales, indicating that higher pain and functional limitation levels are associated with poorer quality-of-life domains. In particular, a strong inverse relationship was identified between the DASH–Work Module and Physical Functioning (r = −0.724, *p* < 0.001), suggesting that upper extremity dysfunction may substantially limit overall physical capacity. Age showed small but statistically significant negative associations with General Health, Physical Functioning, Bodily Pain, and Mental Health subscales (Table 3).

### 3.3. Exploratory Regression Analysis of Factors Affecting Quality of Life

Following the descriptive and bivariate analyses, exploratory regression models were constructed to further describe multivariable patterns of association between clinical variables and health-related quality of life, with each SF-36 subscale treated as a dependent variable and BDI, ZBI, SF-MPQ, DASH, ODI, and age included as independent variables (Table 4). Given the limited sample size relative to the number of predictors, these models were not intended to generate stable, predictive, or generalizable estimates, but rather to describe co-occurring patterns within the present pilot sample. Accordingly, regression outputs are presented strictly as descriptive, hypothesis-generating signals that may inform future confirmatory modeling in adequately powered samples, without implying inferential strength or predictive validity. Although post hoc effect size estimates were reported for descriptive context, the presence of six predictors across multiple regression models with a sample size of n = 40 substantially exceeds commonly recommended observation-to-predictor ratios for stable modeling. Therefore, these analyses should be interpreted strictly as descriptive pattern observations within a feasibility-oriented pilot framework, rather than as statistically reliable, confirmatory, or prediction-capable models. Multicollinearity was examined using variance inflation factor (VIF) and tolerance values, which were within acceptable limits for all predictors (VIF < 10; tolerance > 0.1). Across the exploratory models, depressive symptom severity showed consistent negative associations with several SF-36 subscales, including General Health, Vitality, Role-Emotional, Mental Health, and Bodily Pain. Upper extremity functional limitation (DASH) demonstrated negative associations with Physical Functioning, while caregiver burden (ZBI) showed negative associations with Social Functioning. Low back dysfunction (ODI) was associated with lower General Health scores, and age showed small but statistically significant positive associations with General Health and Vitality domains. Overall, psychological distress, functional limitations, caregiver burden, and age tended to co-occur with reduced quality-of-life domains in caregiving mothers. The exploratory models complement, but do not outweigh, the primary descriptive and bivariate findings presented above. Given the exploratory nature of the analyses, the limited sample size, and the risk of model instability, these findings should be interpreted as descriptive patterns rather than confirmatory or predictive evidence (Table 4).

### 3.4. Comparisons by Physical Activity Level

**Figure 1:**
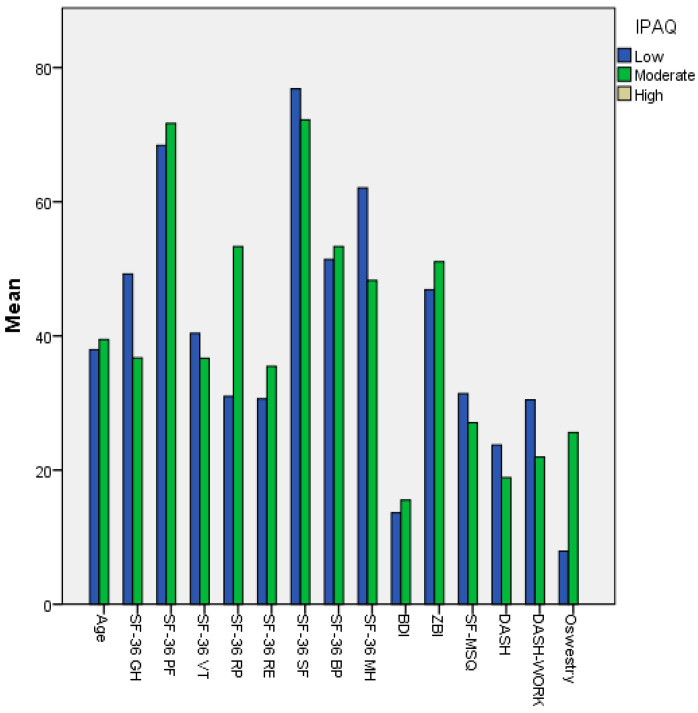
Comparison of physical, psychological, and functional outcomes according to IPAQ physical activity classifications. SF-36 subscales and clinical scores are presented by physical activity category. A *p*-value < 0.05 was considered statistically significant. The categories ‘low’ and ‘moderate’ physical activity are shown, as no participant reported ‘high’ physical activity level.

According to IPAQ classifications, participants with low physical activity levels had significantly higher scores in depression (BDI), pain (McGill), and functional limitation (DASH and ODI), while displaying lower scores in all SF-36 subscales. In contrast, participants with moderate physical activity exhibited significantly higher scores in Vitality, Social Functioning, and Mental Health (*p* < 0.05). These results suggest that higher physical activity levels may be associated with more favorable physical and psychological profiles (Figure 1).

### 3.5. Summary of Descriptive and Exploratory Findings

Across descriptive and bivariate analyses, depression level showed the strongest and most consistent relationship with reduced quality of life. Upper extremity dysfunction and low back pain were associated with notable declines in physical quality of life. Caregiver burden negatively influenced social functioning and emotional well-being.

Physical activity level showed a negative association with depression and fatigue, but a positive association with quality of life.

## 4. Discussion

Our results point to co-occurring relationships between physical and psychological domains in caregiving mothers. Poorer physical health profiles were observed alongside greater emotional distress, and both domains were consistently linked to lower health-related quality of life; however, no directional inference can be made due to the cross-sectional design. These aims were addressed by showing that depression and functional limitation were the variables most strongly associated with reduced quality of life, while higher physical activity levels were associated with better vitality and social functioning. However, given the cross-sectional design, these associations should not be interpreted as directional or causal relationships. Rather than testing predefined causal models, the present pilot findings aim to generate empirically informed hypotheses and to identify clinically meaningful multidimensional patterns that warrant further investigation in larger and longitudinal designs.

Consistent with the results reported, depression showed one of the strongest associations with diminished quality of life across multiple SF-36 subscales. These results are consistent with the findings of Rydzewska et al. (2021), who reported that mothers of children with intellectual disabilities experience greater anxiety, depression, family stress, and emotional burden than mothers of typically developing children [27]. Depressive symptoms consistently co-occurred with lower general health perception, vitality, social functioning, and mental health, underscoring the central role of psychological strain in caregiver well-being. Higher depressive symptom levels were associated with lower social participation, reduced energy levels, and poorer general health perceptions. The results of this study similarly indicate that depressive and anxious symptoms show significant associations with mothers’ general health perceptions and social functioning. These findings confirm our study objectives by demonstrating that both physical and psychological domains contribute significantly to mothers’ health-related quality of life within the caregiving context.

The mean score of the Zarit Caregiver Burden Interview (ZBI) was 48.45 ± 16.02, reflecting a high caregiving burden among participants. This finding aligns with the results of Purpura et al. (2021) and Swai et al. (2024), who noted that higher psychological stress levels exacerbate physical fatigue, which in turn deepens depressive symptoms and further diminishes quality of life [4,5].

Higher caregiver burden was primarily reflected in reduced social functioning and mental health domains, indicating that perceived burden may restrict both emotional resilience and social participation [28].

Child-related characteristics were not incorporated into the present analytical model and should be considered in future research. However the mean participant age of 38.52 years suggests that caregiving has evolved into a chronic physical and psychological load. With increasing age, physical activity levels decreased, while DASH–Work and ODI scores increased. This pattern indicates that prolonged caregiving may lead to cumulative musculoskeletal strain. Similarly, Gokcin Eminel, Kahraman, and Genc (2021) reported that caregiving duration and age are significant contributors to physical pain, fatigue, and chronic musculoskeletal disorders [29].

The mean McGill Pain Questionnaire score of 29.77 ± 10.87 revealed that most participants experienced moderate pain. Pain intensity (SF-MPQ) showed significant inverse associations with selected quality-of-life domains, particularly General Health (r = −0.359, *p* = 0.023), Role Limitations due to Physical Health (r = −0.419, *p* = 0.007), and the SF-36 Bodily Pain subscale (r = −0.442, *p* = 0.004), indicating that higher pain levels are associated with poorer perceived health status and functional role performance. These findings are consistent with Dykens et al. (2014) and Castillo et al. (2022), who reported that pain contributes to functional limitation and psychosocial strain among caregivers [30,31]. Overall, these findings suggest that pain-related functional limitations may coexist with psychosocial strain in caregiving mothers, without implying a direct linear association between pain severity and depressive symptoms.

The DASH and DASH–Work scores indicated that upper extremity dysfunction has a significant impact on physical functioning, daily living activities, and caregiving capacity. Mothers caring for children with physical disabilities often perform repetitive tasks such as lifting, repositioning, and feeding their children. These repeated mechanical loads contribute to fatigue, pain, and restricted mobility in the shoulder and wrist regions. Over time, such strain reduces upper extremity endurance, thereby negatively influence both physical quality of life and general health perception. Similarly, Giray and Akyüz (2019) found significant associations between upper limb dysfunction, reduced grip strength, and increased caregiver burden [32]. These findings underscore the importance of preserving upper extremity function not only for physical health but also for caregiving efficiency and psychological well-being. This perspective is further supported by clinically relevant, locally contextualized evidence linking upper-extremity functional limitation measures (DASH/DASH-W) with health-related quality-of-life domains in a Turkish caregiving sample.

The study also revealed that physical activity level was positively correlated with quality of life, and negatively correlated with depression, pain, and caregiving burden. Mothers who were physically more active demonstrated better psychological and physiological balance. Particularly among those engaging in moderate levels of physical activity, significant improvements were observed in the SF-36 subscales of Vitality, Social Functioning, and Mental Health. Regular physical activity has been widely associated in the literature with improved muscle strength, circulatory efficiency, endurance, and favorable neuropsychological responses, which may relate to lower stress, pain, and depressive symptom levels. These findings indicate that low-threshold, home-based or community-supported physical activity programs may be feasible strategies to improve vitality and mental health in caregiving mothers. As emphasized by Warburton and Bredin (2017), physical activity plays a central role in maintaining biopsychosocial health, with regular exercise significantly improving both cardiovascular endurance and psychological well-being [33]. However, despite the well-established benefits of structured exercise programs, regular participation may be difficult for caregiving mothers due to time constraints, cumulative physical fatigue, continuous caregiving responsibilities, and limited access to organized rehabilitation or exercise facilities, particularly in resource-constrained settings [4,9].

Although cardiometabolic outcomes were not assessed, the co-occurrence of psychological distress and physical inactivity observed here may be clinically relevant because similar profiles have been linked to broader health risks in population studies. Kivimäki et al. (2023) [34] reported that chronic stress disrupts glucose and lipid metabolism, immune responses, and aging processes through the hypothalamic–pituitary–adrenal axis and the autonomic nervous system, which has been discussed as potentially relevant to metabolic dysregulation in population-level research. Similarly, Sakakibara, Obembe, and Eng (2019) found that individuals with multiple cardiometabolic conditions exhibited higher levels of stress and physical inactivity, which were strongly associated with reduced quality of life [35]. Consequently, the coexistence of low physical activity, elevated depression levels, and decreased vitality scores observed in this study may reflect a profile that has been associated in the previous literature with increased cardiometabolic vulnerability. The findings support the notion that chronic stress and psychosocial strain exert not only psychological but also indirect physiological effects through neuroendocrine and metabolic pathways. Although caregiving involves repetitive physical effort, it does not equate to structured aerobic activity; therefore, overall physical activity levels may remain low despite the physically demanding nature of caregiving.

In summary, the results demonstrate that physical and psychological health in mothers of children with physical disabilities form a mutually reinforcing interlinked system. The negative correlations observed between ZBI scores and all SF-36 subscales suggest that caregiving burden contributes not only to physical exhaustion but also to social isolation and emotional burnout. From a clinical perspective, integrated physiotherapy and psychosocial screening may assist in identifying mothers at risk of cumulative physical and emotional strain. Broader policy implications—such as flexible employment arrangements and expanded community support services—extend beyond the empirical scope of this pilot study and should be interpreted as contextual considerations rather than direct findings. These policy-oriented recommendations are consistent with international caregiver support frameworks reported in recent systematic reviews and are conceptually transferable to similar sociocultural contexts [3,27]. Depression, pain, caregiving burden, and physical inactivity appear to form a mutually reinforcing pattern. Because of this cycle, interventions that rely exclusively on structured, center-based exercise programs may have limited feasibility in caregiving populations, whereas flexible, low-threshold approaches such as home-based activity, brief group sessions, and community-supported programs may better accommodate daily caregiving demands and fatigue levels [4,6]. Examples may include free or low-cost community group sessions, brief caregiver-oriented activity counseling integrated into primary healthcare settings, and home-based physiotherapy or activity plans that reduce transportation and scheduling barriers. Breaking this cycle may benefit from the integration of social support mechanisms, psychological counseling, and group-based physiotherapy and exercise programs [36,37]. In practical terms, such approaches may include free or low-cost community-based group exercise sessions, integration of caregiver-oriented physical activity counseling into primary healthcare settings, and home-based physiotherapy or activity programs that reduce transportation barriers and scheduling constraints for caregiving mothers. Strengthening community-based rehabilitation services, expanding home-visit programs, and developing group-based physical activity and psychosocial support interventions may offer culturally feasible and scalable solutions for breaking the cycle of depression, caregiver burden, and physical inactivity within comparable healthcare systems.

Overall, the findings support a multidimensional clinical approach that simultaneously addresses depressive symptoms, pain-related functional limitation, and low physical activity within caregiving populations. Although the observed associations largely align with existing international literature, the present study contributes by contextualizing these multidimensional relationships within a Turkish caregiving population characterized by limited formal support structures and socioeconomic constraints. The combined use of functional, pain-related, psychological, and activity-based measures enables a clinically integrated perspective that may assist clinicians in prioritizing screening domains and tailoring low-threshold interventions in similar settings. As a pilot investigation, the primary contribution lies in refining feasibility, measurement integration, and hypothesis development for later multicenter investigations rather than in generating definitive causal conclusions.

### Limitations

Although effect sizes and multicollinearity indices (VIF and tolerance values) were reported to enhance transparency, the use of multiple regression models with a limited sample size increases the risk of overfitting and unstable estimates. Common statistical heuristics recommend approximately 10–15 observations per predictor for stable multivariable regression modeling; however, the present sample included six predictors with a total sample size of n = 40, corresponding to approximately 6–7 observations per predictor, which may compromise coefficient stability. In addition, the use of multiple correlation analyses and several regression models increases the likelihood of type I error due to multiple testing, particularly in the absence of formal correction procedures (e.g., Bonferroni or false discovery rate adjustment). No adjustment for multiple comparisons was applied, and confidence intervals were not systematically reported for regression coefficients, further limiting the precision and stability of effect size interpretation. Consequently, the reported associations should be viewed as descriptive pilot observations rather than inferentially secure findings.

Because the study employed a cross-sectional design, observed associations cannot be interpreted as causal. Participants were recruited from a single center within the province of Yalova, resulting in a relatively homogeneous socioeconomic profile that limits generalizability to other cultural and demographic settings. In addition, the use of convenience sampling may have introduced selection bias, as mothers who volunteered to participate may differ systematically from those who declined in terms of health status, burden perception, or coping capacity. Child-related variables (e.g., age, sex, type and severity of disability) were not recorded, as the primary focus was maternal physical and psychological burden; however, these characteristics may influence caregiving strain and warrant systematic documentation in larger and more diverse samples.

Data were collected exclusively through self-report questionnaires, which may introduce social desirability bias and subjective reporting error. Physical activity was assessed using the International Physical Activity Questionnaire rather than objective monitoring, which may further affect measurement accuracy. Participant burden represents a methodological trade-off inherent in multidimensional assessment designs. Although the use of multiple validated instruments enhances construct coverage, the administration of 145 items within a single session may introduce fatigue-related measurement error and response bias, potentially affecting data consistency and internal reliability. In caregiving populations characterized by chronic stress and limited discretionary time, prolonged survey administration may influence concentration, attentional endurance, and response reliability. While administration conditions were standardized and short breaks were permitted during pilot feasibility testing, fatigue-related attenuation of response precision cannot be fully excluded. Future studies should evaluate shortened validated instruments or modular assessment strategies to balance measurement breadth with participant sustainability and data quality.

## 5. Conclusions

In conclusion, this pilot study demonstrates that psychological distress, pain-related symptoms, functional limitations, and physical inactivity co-occur with reduced health-related quality of life in caregiving mothers. Depression emerged as the most consistent correlate across quality-of-life domains. As a pilot investigation, these findings should be interpreted as exploratory patterns intended to inform the design of future adequately powered studies.

## Figures and Tables

**Table 2:** Distribution of Demographic Data and Test Scores.

			n = 40	
			Mean ± SD	Median (Min–Max)
Age (Year)			38.52 ± 9.10	36.5 (25.0–68.0)
SF-36 (0–100)	GH		44.55 ± 23.74	38.5 (5.0–85.0)
	PF		69.62 ± 22.76	70.0 (30.0–100.0)
	VT		39.00 ± 17.10	37.5 (10.0–90.0)
	RP		39.37 ± 31.96	43.75 (0.0–100.0)
	RE		32.45 ± 31.55	33.3 (0.0–100.0)
	SF		75.10 ± 22.52	75.0 (12.0–100.0)
	BP		52.12 ± 32.25	53.75 (0.0–100.0)
	MH		56.90 ± 17.07	60.0 (20.0–84.0)
BDI (0–63)			14.35 ± 9.70	12.0 (0.0–42.0)
ZBI (0–88)			48.4 ± 15.8	46 (24.0–88.0)
SF-MPQ (0–45)			29.3 ± 9.7	29.5 (0.0–45.0)
DASH (0–100)			21.91 ± 19.19	17.5 (0.0–90.0)
DASH-W (0–100)			27.26 ± 26.73	22.0 (0.0–75.0)
ODI (0–100)			14.55 ± 15.13	8.0 (0.0–48.0)
			n (%)	
IPAQ		Low	25 (62.5%)	
		Middle	15 (37.5%)	
		High	0 (0%)	
Education		Illiterate	0 (0%)	
		PS	12 (30%)	
		MS	12 (30%)	
		HS	16 (40%)	
		University	0 (0%)	

Data are presented as mean ± standard deviation (SD) and median (minimum–maximum) values. IPAQ: International Physical Activity Questionnaire; SF-36: Short Form-36 Health Survey; PF: Physical Functioning; RP = Role Limitations due to Physical Health; BP: Bodily Pain; GH: General Health; VT: Vitality; SF: Social Functioning; RE: Role Limitations due to Emotional Problems; MH: Mental Health; BDI: Beck Depression Inventory; ZBI: Zarit Caregiver Burden Interview; SF-MPQ: Short-Form McGill Pain Questionnaire; DASH: Disabilities of the Arm, Shoulder and Hand; DASH-W: DASH–Work; ODI: Oswestry Disability Index. The total number of participants is n = 40. According to the IPAQ results, 62.5% of participants had low physical activity levels, 37.5% had moderate levels, and 0% had high levels.

**Table 3:** Analysis of the Relationships Between Test Scores.

			Age	BDI	IPAQ	ZBI	SF-MPQ	DASH	DASH-W	ODI	Education
SF-36	GH	r	−0.324	−0.749	−0.231	−0.336	−0.359	−0.489	−0.546	−0.456	0.019
		p	**0.042**	**0.000**	0.151	**0.034**	**0.023**	**0.001**	**0.000**	**0.003**	0.907
	PF	r	−0.371	−0.545	0.054	−0.292	−0.292	−0.576	−0.724	−0.288	0.129
		p	**0.019**	**0.000**	0.742	0.067	0.067	**0.000**	**0.000**	0.071	0.427
	VT	r	−0.225	−0.685	−0.135	−0.441	−0.225	−0.307	−0.344	−0.331	0.105
		p	0.163	**0.000**	0.407	**0.004**	0.162	0.054	**0.030**	**0.037**	0.519
	RP	r	−0.296	−0.519	0.324	−0.187	−0.419	−0.555	−0.655	−0.083	0.076
		p	0.063	**0.001**	**0.042**	0.249	**0.007**	**0.000**	**0.000**	0.611	0.640
	RE	r	−0.162	−0.458	0.019	−0.291	−0.233	−0.342	−0.293	0.086	−0.142
		p	0.318	**0.003**	0.907	0.069	0.148	**0.031**	0.067	0.598	0.381
	SF	r	−0.083	−0.594	−0.091	−0.604	−0.175	−0.457	−0.435	−0.334	0.047
		p	0.610	**0.000**	0.577	**0.000**	0.281	**0.003**	**0.005**	**0.035**	0.774
	BP	r	−0.337	−0.685	0.023	−0.326	−0.442	−0.584	−0.712	−0.362	0.068
		p	**0.033**	**0.000**	0.886	**0.040**	**0.004**	**0.000**	**0.000**	**0.022**	0.679
	MH	r	−0.330	−0.635	−0.370	−0.563	−0.111	−0.193	−0.248	−0.414	0.065
		p	**0.037**	**0.000**	**0.019**	**0.000**	0.497	0.232	0.123	**0.008**	0.690

r = Pearson correlation coefficient. All previously defined abbreviations apply to this table. A *p*-value < 0.05 was considered statistically significant. Significant negative correlations were observed between several SF-36 subscales and the BDI, ZBI, SF-MPQ, DASH, and ODI scores, with the strongest and most consistent associations involving depression and functional limitation measures. Because higher SF-36 scores indicate better health status, negative correlations reflect poorer quality of life with increasing symptom severity on clinical scales. Overall, depression and functional limitation measures demonstrated the most consistent and pronounced bivariate associations with reduced quality-of-life domains in this sample. Significant results are highlighted in bold.

**Table 4:** Multiple Linear Regression Analysis of Factors Affecting Quality of Life (SF-36 Subscales).

		SF-36							
		GH	RP	VT	RP	RE	SF	BP	MH
Model’s Adjusted R Square		0.690	0.434	0.408	0.386	0.207	0.451	0.607	0.435
Model’s Cohen’s *f*^2^		2.23	0.77	0.69	0.63	0.26	0.082	1.55	0.77
**Age**	p	0.470	**0.047**	0.994	0.306	0.831	0.668	0.284	0.218
	Beta	−0.078	−0.273	0.001	−0.143	−0.034	0.056	−0.120	−0.166
95% CI	LB	−0.766	−1.354	−0.515	−1.486	−1.219	−0.516	−1.219	−0.815
	UB	0.361	−0.010	0.518	0.481	0.986	0.794	0.368	0.193
Collinearity Statistics	Tolerance	0.831	0.831	0.831	0.831	0.831	0.831	0.831	0.831
	VIF	1.203	1.203	1.203	1.203	1.203	1.203	1.203	1.203
**BDI**	p	**<0.001**	0.274	**0.002**	0.051	**0.035**	0.289	**0.006**	**0.036**
	Beta	−0.600	−0.200	−0.617	−0.384	−0.468	−0.191	−0.439	−0.392
95% CI	LB	−2.190	−1.328	−1.749	−2.523	−2.932	−0.1281	−2.474	−1.335
	UB	−0.749	0.390	−0.429	−0.009	−0.113	0.393	−0.446	−0.046
Collinearity Statistics	Tolerance	0.448	0.448	0.448	0.448	0.448	0.448	0.448	0.448
	VIF	2.232	2.232	2.232	2.232	2.232	2.232	2.232	2.232
**ZBI**	p	0.370	0.966	0.592	0.596	0.572	**0.007**	0.743	0.056
	Beta	0.908	0.006	−0.082	0.082	−0.099	−0.417	0.041	−0.292
95% CI	LB	−0.201	−0.423	−0.421	−0.467	−0.909	−1.017	−0.428	−0.641
	UB	0.525	0.442	0.244	0.800	0.511	−0.174	0.594	0.008
Collinearity Statistics	Tolerance	0.670	0.670	0.670	0.670	0.670	0.670	0.670	0.670
	VIF	1.492	1.492	1.492	1.492	1.492	1.492	1.492	1.492
**SF-MPQ**	p	**0.047**	0.828	0.240	0.276	0.522	0.652	0.055	0.752
	Beta	−0.228	−0.030	−0.168	−0.207	−0.468	−0.062	−0.439	−0.044
95% CI	LB	−1.109	−0.727	−0.801	−1.646	−2.932	−0.783	−2.474	−0.569
	UB	−0.008	0.586	0.208	0.276	−0.113	0.497	−0.446	0.415
Collinearity Statistics	Tolerance	0.770	0.770	0.770	0.770	0.770	0.770	0.770	0.770
	VIF	1.298	1.298	1.298	1.298	1.298	1.298	1.298	1.298
**DASH**	p	0.306	**0.005**	0.693	0.056	0.640	0.081	**0.030**	0.635
	Beta	−0.126	−0.454	0.061	−0.311	−0.084	−0.267	−0.260	0.072
95% CI	LB	−0.461	−0.902	−0.225	−1.051	−0.736	−0668	−1.641	−0.209
	UB	0.149	−0.174	0.334	0.014	0.459	0041	−0.090	0.337
Collinearity Statistics	Tolerance	0.638	0.638	0.638	0.638	0.638	0.638	0.638	0.638
	VIF	1.568	1.568	1.568	1.568	1.568	1.568	1.568	1.568
**ODI**	p	**0.035**	0.279	0.632	0.605	0.060	0.345	0.121	0.270
	Beta	−0.238	−0.148	−0.067	0.073	0.311	−0.127	−0.179	−0.151
95% CI	LB	−0.720	−0.636	−0.393	−0.449	−0.030	−0.592	−0.869	−0.481
	UB	−0.027	0.190	0.242	0.759	1.325	0.213	0.106	0.139
Collinearity Statistics	Tolerance	0.797	0.797	0.797	0.797	0.797	0.797	0.797	0.797
	VIF	1.255	1.255	1.255	1.255	1.255	1.255	1.255	1.255

The dependent variables are the SF-36 subscales. The independent variables are BDI, ZBI, SF-MPQ, DASH, ODI, and Age. A *p*-value < 0.05 was considered statistically significant. Significant results are highlighted in bold.

## Data Availability

The data presented in this study are available on request from the corresponding author due to ethical and privacy restrictions.
